# Prevalence, Risk Factors, and Antimicrobial Susceptibility Patterns of *Salmonella* From Bulk Milk at the Dairy Farm Level in Mekelle and Southeast Zones of Tigrai, Ethiopia

**DOI:** 10.1002/mbo3.70190

**Published:** 2025-11-30

**Authors:** Atsebaha Gebrekidan Kahsay, Tsehaye Asmelash, Enquebaher Kassaye

**Affiliations:** ^1^ Department of Medical Microbiology and Immunology, College of Health Sciences Mekelle University Mekelle Tigrai Ethiopia; ^2^ Department of Veterinary Public Health and Food Safety, College of Veterinary Sciences Mekelle University Mekelle Tigrai Ethiopia

**Keywords:** antimicrobial susceptibility patterns, bulk milk, Ethiopia, Mekelle, Salmonella, Tigrai

## Abstract

*Salmonella* is recognized as one of the foodborne bacterial infections. The bacterium spreads through contact with animals and ingestion of contaminated foods. This study aimed to determine the prevalence, risk factors, and antimicrobial susceptibility patterns of *Salmonella* from bulk milk at dairy farm level in Mekelle and Southeast Zones of Tigrai, Ethiopia. A cross‐sectional study was carried out from January to June 2025. After taking the consent, sociodemographic, risk factors, and 203 bulk milk samples were collected from the dairy farms. *Salmonella* was isolated and identified through pre‐enrichment, selective enrichment, selective media, and a series of biochemical tests. Antimicrobial susceptibility testing was conducted using the disk diffusion method. Stata v‐16 was employed to determine the strength of the factors that associates with *Salmonella*. The prevalence of *Salmonella* was six (2.96%). *Salmonella* positivity showed statistically significant association with farms that don't practice regular udder washing before milking, lack of knowledge about bacterial infections, and do not know that consumption of raw milk cause foodborne illness. Five (83.3%) isolates of *Salmonella* showed resistance to ampicillin and tetracycline, and four (66.7%) to streptomycin. All *Salmonella* isolates were susceptible to ceftazidime and cefotaxime. Three isolates of *Salmonella* showed multidrug resistance. The prevalence of *Salmonella* was low, but its presence in milk may be considered a potential risk to milk safety. Three *Salmonella* isolates showed resistance to four and six antimicrobial classes. The finding highlights the need for appropriate hygiene practices and the correct use of antibiotics in the farms.

## Graphical abstract

Graphical Abstract test on Prevalence, Antimicrobial Susceptibility Patterns and Associated Factors of Salmonella from Bulk Milk at Dairy Farm Level in Mekelle and Southeast Zones of Tigrai, Northern Ethiopia.

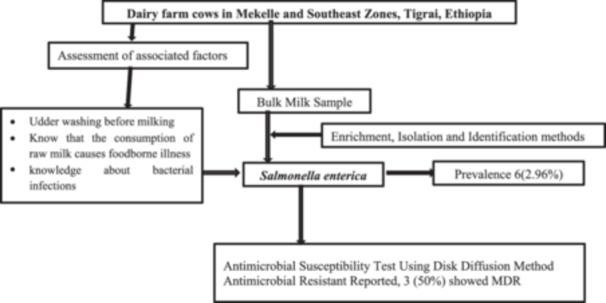

## 1 Background

Foodborne infections are a major public health issue in both developing and developed nations. Every year, more than 600 million foodborne infections with 420,000 fatalities are reported globally (WHO 2022a). Nowadays, over 250 different foodborne infections have been recognized, and most of them are pathogens caused by bacteria. These are commonly found in animals and animal products, including milk, meat, cheese, and yoghurt (CDC 2018).

Cow milk has high water activity and nutritive value, which serves as a kind of medium for the growth of microorganisms (WHO 2022b; Duguma 2022). Microorganisms commonly identified in milk and its products can cause significant human illnesses. The most common bacteria that cause foodborne infections are *Campylobacter spp., Salmonella enterica, Escherichia coli O157: H*, and *Listeria monocytogenes*. One of them is *Salmonella*, which accounts for the major part of foodborne infections (CDC 2018).

*Salmonella enterica* are gram‐negative rod‐shaped facultative anaerobic bacteria that possibly infect a wide range of hosts. The bacteria comprised two species, *Salmonella bongori* and *Salmonella enterica (*Feasey et al. 2012 *)*. *Salmonella enterica* has six subspecies, which are enterica, salamae, arizonae, diarizonae, houtenae, and indica, with over 2,600 serotypes. Among these, nearly 60% are contributed by *Salmonella enterica* subspecies *enterica*, and 99% of the subspecies can cause illness in humans and animals (Ferrari et al. 2019). *Salmonella* Typhi and *Salmonella* Paratyphi, limited to human reservoirs, cause typhoid and paratyphoid fever, respectively; whereas *Salmonella* Typhimurium and *Salmonella* Enteritidis, dominant causes of zoonoses, have multiple hosts (Crump et al. 2015).

Milk produced for human consumption should not have any infectious agents, although milk and its products, particularly raw or unpasteurized milk, are considered a vehicle for the transmission of *Salmonella* infection to humans (Gebeyehu et al. 2022; Asfaw et al. 2022; WHO 2018). The source of milk contamination by *Salmonella* comes from the feces of infected cattle, contaminated milkers' hands, contaminated udder, and contaminated milking equipment during milking, processing, transportation, and storage (Vairamuthu et al. 2010). Although contamination of milk by *Salmonella* was reported, consumption of raw or unpasteurized milk is still in practice (Diniso and Jaja 2024). This may be due to a lack of awareness on the safe handling of milk and the risk of infectious agents (Lindah et al. 2018; Deneke et al. 2022), as well as the perception of fear of losing the nutritional value of the milk while boiling, as reported from a study in Ethiopia (Amenu et al. 2019).

Consumption of milk contaminated with *Salmonella* bacteria exposes human beings to salmonellosis (Van Kessel et al. 2013; BLAU et al. 2005), which may have serious health (Pieracci et al. 2016) and economic impact (Mekonnen et al. 2021). Salmonellosis is one of the four key global causes of diarrheal diseases, and its clinical outcomes are acute fever, diarrhea, nausea, vomiting, and abdominal pain. Due to the typically mild symptoms of salmonellosis, most patients recover without treatment. However, the resulting dehydration can occasionally become severe and potentially fatal, especially in young patients and elderly individuals (WHO 2018).

The rates of *Salmonella* prevalence among milk and milk products were reported from different areas of the world including Italy (0.1%) (Busani et al. 2005), Spain (0%) (CABEDO et al. 2008), Ecuador (37.5%) (Loor‐Giler et al. 2025), Mongolia (14.26%) (Xie et al. 2025) and in different locations of Ethiopia including Somalia‐Ethiopia (3.3%) (Reta et al. 2016), Addis Ababa (3.1%) (Addis et al. 2011), Oromia (2%) (Temesgen et al. 2025), Oromia (1.2%) (Aliyo et al. 2022), Oromia (3.9%) (Hunduma et al. 2024), Wolaita Sodo (1.89%) (Ayichew et al. 2024), Sidama region (1.85%) (Geinoro et al. 2025), Oromia, Amhara and Southern Ethiopia (19.7%) (Bedassa et al. 2023), Gondar (21.9%) (Yirsa and Tigistu 2025), South Ethiopia (10.42%) (Gebeyehu et al. 2022), Wolaita Soda (9.3%) (Asefa et al. 2023), Oromia (14%) (Gume et al. 2023), and Tigrai (18.8%) (Abebe et al. 2014).

The risks and costs of antimicrobial resistance (AMR) around the globe are broadly documented. For instance, antibiotic resistance pathogens cause about 33,000 annual deaths in Europe. Moreover, 4.95 million total deaths cause 1.5 billion EUR in healthcare expenses and productivity losses annually (Caneschi et al. 2023). One of the bacterial pathogens that became resistant to commonly used antibiotics is *Salmonella enterica*. The bacteria showed resistance to ampicillin, streptomycin, and chloramphenicol (Gebeyehu et al. 2022; Van Kessel et al. 2013; Addis et al. 2011; Abebe et al. 2014) with no resistance to ciprofloxacin (Van Kessel et al. 2013; Addis et al. 2011).

Implementing good hygiene on dairy farms and ensuring the safety of their food products based on the scientific standards and protocols provided are advantages for consistent productivity on dairy farms and ensuring the health of the public and the community in Tigrai regional State of Ethiopia. Continuous surveillance of *Salmonella* and its antimicrobial resistance pattern in dairy farms is paramount in designing prevention and control measures of the diseases. Hence, the objectives of the study were to determine the prevalence, risk factors, and antimicrobial susceptibility patterns of *Salmonella* from bulk milk at the dairy farm level in Mekelle and the Southeast zones of Tigrai, Ethiopia.

## 2 Methods and Materials

### 2.1 Study Area

**Figure 1:**
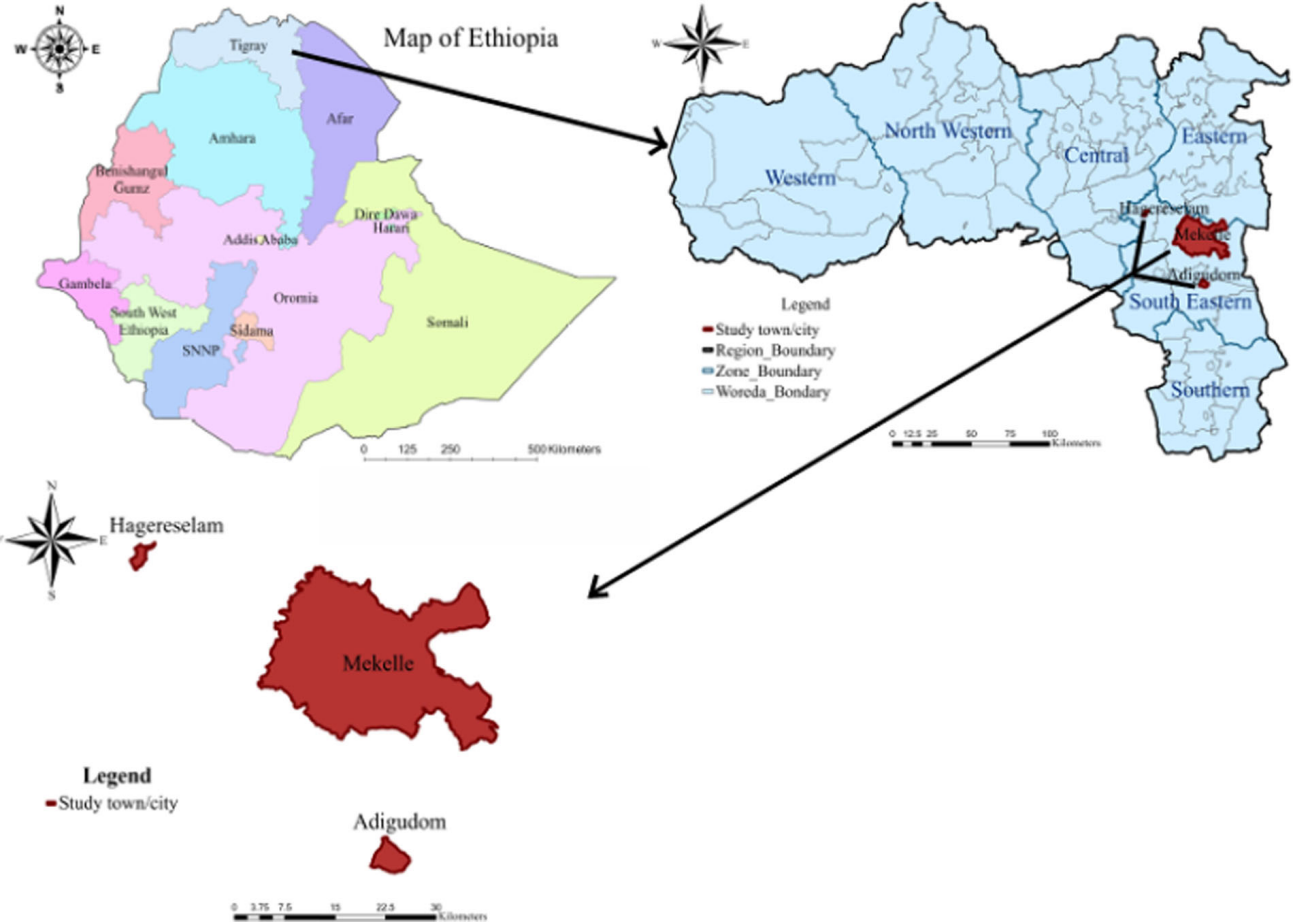
A map showing study sites for prevalence, risk factors, and antimicrobial susceptibility patterns of *Salmonella* from Bulk Milk at Dairy Farm Level in Mekelle and Southeast Zones of Tigrai, Ethiopia, January to June 2025, Adopted from Kahsay et al, 2025 (Kahsay et al. 2025).

The Tigrai Region of Ethiopia lies between 12°14'50.50″ and 14°53'48.03″ N latitude and 36°26'48.74″ and 39°59'0.09″ E longitude. The South East Zone of Tigrai, one of the region's seven administrative zones, is bordered by the Southern Zone to the south, the Amhara Region to the southeast, the Central Zone to the northeast, the East Zone to the north, and the Afar Regional State to the east, and it surrounds the Mekelle Special Zone. According to the 2019/20 Central Statistical Agency (CSA) report (Federal Democratic Republic of Ethiopia Central Statistical Agency 2021), the Tigrai Regional State has over 4.9 million cattle, more than 171,000 dairy farms, and about 778,000 milking cows, producing over 209 million liters of milk annually. Mekelle comprises seven sub‐cities, while the South East Zone includes five towns. The study was carried out in Mekelle and the Southeast Zones of Tigrai, Ethiopia, as shown in Figure 1. These areas were selected due to budget limitations, the absence of Salmonella‐related studies on dairy farms over the past 13 years, and the relatively high density of dairy farms in these locations.

### 2.2 Research Design, Study Period, and Study Population

A cross‐sectional study was carried out from January to June 2025. Bulk milk samples were collected from the dairy farms of Mekelle and the Southeast zone of Tigrai, Ethiopia.

### 2.3 Sample Size Determination

The sample size was calculated based on a single population proportion formula using the previous study conducted in the Tigrai, Ethiopia, 13 years ago with a prevalence rate of 18.8% (Abebe et al. 2014) at a 95% confidence interval with a 5% absolute precision (*d*).

$$\frac{n={\left(Z\alpha {/}_{2}\right)}^{2}*P(1-P)}{d^{2}}=\frac{{\left(1.96\right)}_{*}^{2}0.188(1-0.188)}{{\left(0.05\right)}^{2}}=235$$

*n* = sample size, *Z* = Z statistic stands for a level of confidence, *P* expected prevalence or proportion, *d* = precision.

The overall dairy farms in both the study areas was 1161 (Sources: Mekelle and South East Zone agricultural office), and we corrected the sample size using the population correction formula

$$\text{NC}=n_{0}/1+\left(n_{0}-1\right)/N={\text{Nn}}_{o}/N+\left(n0-1\right)=1161*235/1161+\left(235-1\right)=196+4\%\;\text{contingency}\left(7\right)=203$$

NC = Corrected sample size with contingency (NC = 203)

*n*_0_ = Initial sample size (*n*_o_=235)

*N* = Total population size (1161), which is the available population in the three sub‐cities and two towns

$$\text{Population}\;\text{proportion}\;\text{allocation}=\frac{\text{Population}\;\text{in}\;\text{each}\;\text{sub}\;\text{city}\;\text{or}\;\text{town}}{\text{Total}\;\text{population}\;\text{in}\;\text{two}\;\text{areas}}*\text{corrected}\;\text{sample}\;\text{size}$$

Accordingly,

Ayder (204/1161*203) = **35**; Semen (297/1161 * 203) = **52**; Hawelti (385/1161*203) = **67**; Adigudem (66/1161*203) = **12**; Hagereselam (215/1161*203) = **37**.

### 2.4 Sampling Method

A stratified random sampling approach with proportional allocation was applied, in which the sample size for each site in the Southeast and Mekelle zones was determined according to its proportion of the overall target population. Random selection within each site was carried out using a lottery method.

### 2.5 Survey Data Collection

Dairy farms located in Mekelle and the Southeast zones are the main sources of milk for Mekelle city's cafeterias, restaurants, hotels, as well as household consumers in the city. A regular survey of dairy farms related to hygiene and safety of milk is important to prevent the transmission of microbial pathogens, including *Salmonella*, and to overcome unexpected foodborne outbreaks in the study area. A structured questionnaire related to dairy farms was prepared and translated into the local language, Tigrigna. It was approved by the Institutional Review Board of the Mekelle University College of Health Sciences (**MU‐IRB 2439/2024**). The survey was conducted using Kobo Toolbox through face‐to‐face interviews with dairy farm personnel involved in milking and cleaning activities, following the completion of pretests. Food safety training, presence of a separate milking house, sources of water for the cleaning of milk containers, regular udder washing before milking, regular hand washing before milking, mixing of milk collected from the diseased animal with that of the healthy one, antiseptic use before and after milking are some of the questions included in the survey. Aside from providing a questionnaire, a direct examination of the general cleanliness and sanitary practices of the dairy farms was conducted and recorded. The survey was administered at the time of bulk milk sample collection for *Salmonella* identification.

### 2.6 Milk Sample Collection

A sterile screw‐capped bottle was used to collect approximately 30 ml of bulk milk sample from each dairy farm. All samples were labeled with the date of sampling, type of sample, and the name of the farms. Milk samples were collected and transported using a cold chain at 4°C within 4 h of collection to the Microbiology laboratory at College of Health Sciences, Mekelle University, and Tigrai Region, Northern Ethiopia. Sample collection, isolation, and identification procedures were performed based on the World Health Organization Global Foodborne Infections Network protocol (WHO Global Foodborne Infections Network 2010) and ISO 6579‐1 (ISO 2017).

### 2.7 Bacteriological Examination of *Salmonella enterica*

*Salmonella* isolation and identification were performed using buffered peptone water (BPW, HIMEDIA, India) for pre‐enrichment, followed by enrichment in Rappaport–Vassiliadis broth and Selenite Cysteine broth (HIMEDIA, India). Selective plating was carried out on Xylose Lysine Deoxycholate agar and Brilliant Green agar (HIMEDIA, India), and the isolates were subsequently confirmed through a series of biochemical tests.

### 2.8 Nonselective Pre‐Enrichment Media

The bulk milk samples were pre‐enriched in BPW in the ratio of 1:9 (i.e., 25 mL of the sample was inoculated into 225 mL of BPW) and incubated at 37°C for 24 h.

### 2.9 Selective Enrichment Media

Selective enrichment was carried out using Rappaport–Vassiliadis medium (RV, HIMEDIA, India) and Selenite Cysteine broth (HIMEDIA, India). Portions of the pre‐enriched bacterial growth were inoculated into enrichment medium and incubated under the appropriate conditions for *Salmonella* recovery.

### 2.10 Isolation and Identification

For selective isolation, cultures from the enrichment media were plated onto Xylose Lysine Deoxycholate (XLD; HIMEDIA, India) agar and Brilliant Green Agar (BGA; HIMEDIA, India). After incubation, plates were evaluated for characteristic *Salmonella* colony appearances—black‐centered colonies, with a reddish translucent zone on XLD, and red to pink‐white opaque colonies, with a surrounding red halo on BGA. Representative typical or suspicious colonies were sub‐cultured onto nutrient agar (HIMEDIA, India) in line with ISO 6579‐1 (ISO 2017). Non‐lactose‐fermenting colonies were subsequently examined using standard biochemical media, including TSI, Simmons citrate, urea, lysine iron, and SIM formulations. Colonies exhibiting biochemical profiles consistent with *Salmonella,* such as characteristic sugar reactions, hydrogen sulfide production, lysine decarboxylation, urea negativity, indole negativity, and citrate utilization, were identified as *Salmonella*. Confirmed isolates from bulk milk samples were then used for antimicrobial susceptibility assessment.

### 2.11 Antimicrobial Susceptibility Patterns

*Salmonella* isolates obtained from bulk milk samples on dairy farms were tested for antimicrobial susceptibility using the Kirby–Bauer disk diffusion method, following Clinical and Laboratory Standards Institute (CLSI) guidelines (Clinical and Laboratory Standard Institute 2024). In summary, single colonies of *Salmonella* grown on nutrient agar were collected with a wire loop and transferred into a tube containing 5 mL of sterile normal saline, then emulsified. The suspension was incubated at 37°C until it reached the 0.5 McFarland turbidity standards. Using a sterile cotton swab, the standardized inoculum was evenly spread over the surface of Mueller–Hinton agar plates inside a biosafety cabinet. The plates were allowed to dry at room temperature for 15 min before applying antibiotic disks of known concentrations. After placing the disks, the plates were incubated at 36°C for 18 h.

Twelve antimicrobial disks representing nine classes were obtained from HIMEDIA (India). The tested antimicrobials included Penicillins: ampicillin (AMP, 10 µg); Penicillin/β‐lactam inhibitor combination: amoxicillin–clavulanic acid (20/10 µg); Amphenicoles: chloramphenicol (C, 30 µg); Fluoroquinolones: ciprofloxacin (CIP, 5 µg); Folate pathway inhibitors: cotrimoxazole (Cot, 25 µg); Cephalosporins: ceftriaxone (CRO, 30 µg), cefotaxime (CTX, 30 µg), and ceftazidime (CTZ, 30 µg); Tetracyclines: tetracycline (TT, 30 µg); Carbapenems: meropenem (MER, 10 µg); and Aminoglycosides: streptomycin (S, 10 µg) and gentamicin (GEN, 10 µg). Following incubation, the inhibition zone diameters were measured to the nearest millimeter using a calliper. Interpretation of susceptibility: categorized as susceptible (S), intermediate (I), or resistant (R) and was carried out according to CLSI breakpoints (Clinical and Laboratory Standard Institute 2024).

**Figure 2:**
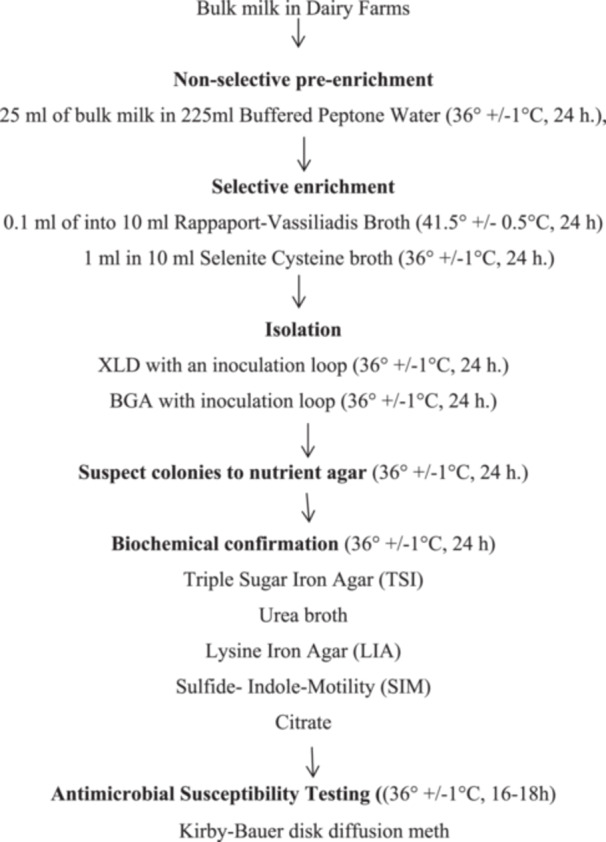
A flow diagram for laboratory diagnosis of *Salmonella* in bulk milk samples collected from Mekelle and Southeast Zones of Tigrai, Ethiopia (WHO Global Foodborne Infections Network (WHO Global Foodborne Infections Network 2010)).

The workflow for pre‐enrichment, enrichment, isolation, identification, and antimicrobial susceptibility test was illustrated in Figure 2.

### 2.12 Quality Management and Data Analysis

Before conducting the investigation, all equipment used for sample processing was thoroughly checked. Quality control was ensured by using *E. coli* (ATCC 25922) as the negative control and *Salmonella* Typhimurium (ATCC 14028) as the positive control. Data were coded, entered, and stored in SPSS version 23, while Fisher's exact test was carried out in Stata version 16 to examine the association between *Salmonella* prevalence and potential risk factors. Multivariate logistic regression was applied to identify variables significantly linked with *Salmonella* detection. Statistical significance was defined at a 95% confidence level with a *p*‐value < 0.05.

### 2.13 Ethical Clearance

An ethical clearance letter was collected from the Institutional Review Board of the College of Health Sciences, and its Institutional Review Board (IRB) number is **MU‐IRB 2439/2024**. A support letter was collected from Mekelle University College of Veterinary Sciences. Once assured the informed consent from the dairy farm owners and assent from the guardians of the participants who did not write or read, survey data were collected from Mekelle and Southeast Zones of Tigrai, Northern Ethiopia.

## 3 Results

### 3.1 Background Characteristics of Humans Having Contacts With Dairy Farms

A total of 203 bulk milk samples were collected from 203 dairy farms, one bulk milk sample from each farm, attaining a 100% response rate. The ages of 65 (32%) of the humans in contact with the dairy farms were between 31 and 40 years old, and the majority, 146 (71.9%) of the humans in contact with dairy farms were males. The proportion of dairy farms in Mekelle and the Southeast zones of Tigrai was 154 and 49, respectively. The highest, 67 (33%), and the lowest, 12 (5.9%) proportions of the dairy farms included in the study were Hawelti subcity, Mekelle zone, and Adigudem town from the Southeast Zone of Tigrai. More than two‐thirds, 140 (69%) of the dairy farms were established between 2011 and 2020 (Table 1).

**Table 1:** Background characteristics of humans in contact with the dairy farms of mekelle and Southeast Zones of Tigrai, Ethiopia, January to June 2025 (*N* = 203).

Variables	Frequency	Percent
**Age in years**		
20‐30	34	16.7
31‐40	65	32.0
41‐50	43	21.2
51‐60	34	16.7
> 60	27	13.3
**Gender**		
Male	146	71.9
Female	57	28.1
**Study sites**		
Ayder	35	17.2
Semen	52	25.6
Hawelti	67	33.0
Hagereselam	37	18.2
Adigudem	12	5.9
**Dairy Farm Eastablishment**		
2000 and before	14	6.9
2001 to 2010	24	11.8
2011–2020	140	69.0
2021 and after	25	12.3
	Total	203

### 3.2 Hygiene Practices in the Dairy Farms of Mekelle and Southeast Zones of Tigrai, Ethiopia

The safety and hygienic aspects of the dairy farms located in Mekelle and the Southeast zones of the Tigrai region were investigated in this study. The humans in contact with the dairy farms that were involved in food safety training, those who did not have separate milking houses and use of well water for cleaning of milk containers were 25 (12.3%), 190 (93.6%), and 81 (39.9%), respectively. Regular udder washing and regular hand washing before milking were not performed in 47 (23.2%) and 11 (5.4%) dairy farms, respectively. The respondents who do not have knowledge about bacterial infections and do not know that consumption of raw milk causes foodborne illness were 107 (52.7%) and 70 (34.5%), respectively (Table 2).

**Table 2:** Frequency of hygiene practices in the dairy farms of mekelle and Southeast Zones of Tigrai, Ethiopia, January to June 2025 (*N* = 203).

Variables	Frequency	Percent
**Have you ever attended food safety training**		
Yes	25	11.8
No	178	88.2
**Presence of Separate Milking House**		
Yes	13	6.4
No	190	93.6
**Sources of Water to Clean Milk Containers**		
Tap water	122	60.1
Wells	81	39.9
**Udder Washing before milking**		
Yes	156	76.8
No	47	23.2
**Hand Washing before Milking**		
Yes	192	94.6
No	11	5.4
**Antiseptic Use before and after Milking**		
Yes	56	27.6
No	147	72.4
**Do you filter milk**		
Yes	192	94.6
No	11	5.4
**Knowledge about Bacterial infection**		
Yes	96	47.3
No	107	52.7
**Type of milk container**		
Plastic	193	95.1
Stainless steel	10	4.9
**Do you think consumption of raw milk causes foodborne illness?**		
Yes	133	65.5
No	70	34.5
**Availability of hygiene protocol to prevent bacterial contamination**		
Yes	12	5.9
No	191	94.1

### 3.3 Prevalence of *Salmonella* by Study Sites in Mekelle and Southeast Zones of Tigrai, Ethiopia

The overall prevalence of *Salmonella* in the current study was six (2.96%). The prevalence rates of *Salmonella* in Ayder and Semen subcities were 2 (5.7%) and 2 (3.8%), respectively. Similarly, the prevalence of *Salmonella* in Adigudem and Hagereselam was 0 (0%) and 1 (2.7%), respectively (Table 3).

**Table 3:** Prevalence of *Salmonella* by study sites in Mekelle and Southeast Zones of Tigrai, Ethiopia, January to June 2025.

Variables	*Salmonella* isolates		Total (*n* = 203)
	*Salmonella* positive, *N* (%)	*Salmonella* Negative, *N* (%)	
Sub Cities/Towns			
Ayder	2 (5.7)	33 (94.3)	35 (17.2)
Semen	2 (3.8)	50 (96.2)	52 (25.6)
Hawelti	1 (1.5)	66 (98.5)	67 (33)
Hagereselam	1 (2.7)	36 (97.3)	37 (18.2)
Adigudem	0 (0)	12 (100)	12 (5.9)
Total	6 (2.96)	197 (97)	203 (100)

### 3.4 Fisher's Exact Test of Risk Factors Associated With *Salmonella* in Mekelle and Southeast Zones

All *Salmonella* isolates were recovered exclusively from the bulk milks of dairy farms that do not have a separate milking house (p‐V = 1.00) and do not have specific hygiene protocols (p‐V = 1.00) in Fisher's exact test. No *Salmonella* isolates were identified from the bulk milk of the dairy farms that do have a separate milking house and specific hygiene protocols. *Salmonella* positivity showed statistically significant association with humans who do not practice regular udder washing before milking (p‐V = 0.027), do not have knowledge about bacterial infection (p‐V = 0.030), and those who do not know that consumption of raw milk causes foodborne illness (p‐V = 0.019), (Table 4).

**Table 4:** Fisher's exact test for factors associated with *Salmonella* in bulk milk of dairy farms in Mekelle and Southeast Zones of Tigrai, Ethiopia, January to June 2025 (*N* = 203).

Variable	*Salmonella* Negative		*Salmonella* Positive		*p* value
	*n*	%	*n*	%	
**Has anyone ever attended food safety training?**					
Yes	24	96.0	1	4.0	0.550
No	173	97.2	5	2.8	
**Presence of a separate milking house**					
Yes	13	100.0	0	0.0	1.000
No	184	96.8	6	3.2	
**Sources of water for the cleaning of milk containers**					
Tap water	120	98.4	2	1.6	0.219
Wells	77	95.1	4	4.9	
**Regular udder washing before milking**					
Yes	154	98.7	2	1.3	0.027
No	43	91.5	4	8.5	
**Regular hand washing before milking**					
Yes	187	97.4	5	2.6	0.287
No	10	90.9	1	9.1	
**Mixing of milk collected from the diseased animal with that of the healthy one**					
Yes	33	94.3	2	5.7	0.276
No	164	97.6	4	2.4	
**Antiseptic use before and after milking**					
Yes	53	94.6	3	5.4	0.350
No	144	98.0	3	2.0	
**Do you filter milk**					
Yes	187	97.4	5	2.6	0.287
No	10	90.9	1	9.1	
**Knowledge about bacterial infections**					
Yes	96	100.0	0	0.0	0.030
No	101	94.4	6	5.6	
**Do you think consumption of raw milk causes foodborne illness?**					
Yes	132	99.2	1	0.8	0.019
No	65	92.9	5	7.1	
**Do you have a specific hygiene protocol in place to prevent bacterial contamination**					
Yes	12	100.0	0	0.0	1.000
No	185	96.9	6	3.1	

### 3.5 Multivariable Logistic Regression Analysis of Factors Associated With *Salmonella*

Because of the small number of positive isolates, traditional logistic regression was not practical. Firth's logistic regression (penalized logistic regression) was employed to model the relationship between *Salmonella* positivity and associated factors. This technique minimizes small‐sample bias and yields estimates of the finite odds ratio. It is important to view the results as exploratory due to the limited number of incidents (Suhas et al. 2023). This was done using Stata 16 to identify independent predictors. All *Salmonella* positive results were found in the bulk milk samples collected from the dairy farms that do not have a separate milking house, do not have knowledge about bacterial infection, and do not have a hygiene protocol. There were no *Salmonella* isolates recovered from the bulk milk samples collected from the dairy farms that responded “yes”. Due to this, these variables were not included in the multivariable analysis. As a result, a multivariable analysis composed of two variables was fitted. The adjusted odds of positive *Salmonella* were 5.44 times higher among those who don't wash udder before milking regularly (AOR = 5.44; 95% CI: 1.09 to 27.17; p‐V = 0.039). Likewise, the adjusted odds of positive *Salmonella* isolates were 6.43 times higher among those who don't know that consumption of raw milk causes food‐borne illness (AOR = 6.43; 95% CI: 1.01 to 40.70; p‐V = 0.048), Table 5.

**Table 5:** Multivariable logistic regression analysis of factors associated with *Salmonella* in bulk milk of dairy farms in mekelle and Southeast Zones of Tigrai, Ethiopia, January to June 2025.

Predictors	COR [95% CI]	*p* value	AOR [95% CI]	*p* value
**Regular udder washing before milking**				
Yes	1		1	
No	6.39 [1.31, 31.11]	0.022	5.44 [1.09, 27.17]	0.039
**Know that the consumption of raw milk causes foodborne illness.**				
Yes	1		1	
No	7.42 [1.19, 46.22]	0.032	6.43 [1.01, 40.70]	0.048

### 3.6 Antimicrobial Susceptibility Patterns of *Salmonella*

**Figure 3:**
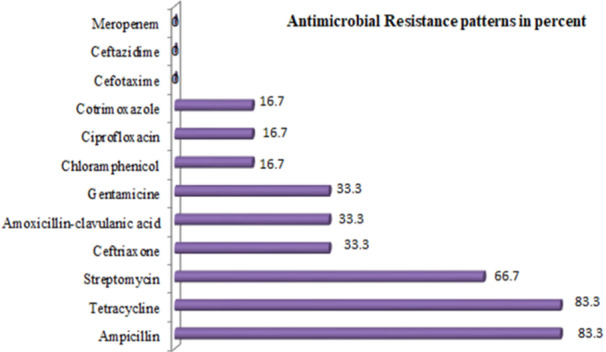
Antimicrobial susceptibility patterns of salmonella from bulk milk in dairy farms of Mekelle and Southeast Zones of Tigrai, Ethiopia, January to June 2025.

Overall, 12 antimicrobial disks from nine classes were tested to six *Salmonella* isolates. The resistance patterns of *Salmonella* isolates to ampicillin, tetracycline, and streptomycin were 5 (83.3%), 5 (83.3%), and 4 (66.7%), respectively. There was no isolate of *Salmonella* that showed resistance to any of the two cephalosporin category (ceftazidime, cefotaxime) and to meropenem, Figure 3.

### 3.7 Multidrug‐Resistant Isolates of *Salmonella* From Bulk Raw Cow Milk

Multidrug resistance (MDR) is defined as resistance of a bacterial isolate to at least three different classes of antimicrobial agent, according to the criteria described by Magiorakos et al in 2012 (Magiorakos et al. 2012). The overall MDR isolates of *Salmonella* in this study were 3 (50%). The MDRs presented in the current study showed that one to three, one to four, and one to six antimicrobial classes, Table 6.

**Table 6:** Multidrug‐resistant isolates of *Salmonella* from bulk milk of dairy farms in Mekelle and Southeast Zones of Tigrai, Ethiopia, January to June 2025.

Antibiotics	Frequency	Antibiotic classes	Total MDR
Amp	1	1	—
T, S	1	2	—
AMC, AMP, T	1	2	—
AMP, T, S	1	3	1
AMC(AMP), CRO, T, S(G)	1	4	1
AMP, CIP, COT, CRO, T, S(G)	1	6	1
Total	6		3 (50%)

Abbreviations: AMC = Amoxacillin/clavulanic acid, AMP = Ampicillin, CIP = Ciprofloxacin, COT = Cotrimoxazole, CRO = Ceftriaxone, G = Gentamicine, S = Streptomycin, T = Tetracycline.

## 4 Discussion

The primary carriers of *Salmonella* enterica are animals that have the capacity to transmit the infection to humans through intimate contact (Hoelzer et al. 2011) and the consumption of animal‐source foods, such as raw milk (Galán‐Relaño et al. 2023). The overall prevalence of *Salmonella* in our study was six (2.96%), which is similar to studies reported from cow milk in different regions of Ethiopia, including Oromia (3.9%) (Hunduma et al. 2024), Ethiopia‐Somalia (3.3%) (Reta et al. 2016), Addis Abeba (3.1%) (Addis et al. 2011). However, our finding was lower than another studies conducted on cow milk and its products in many areas of Ethiopia including Southern Ethiopia (10.42%) (Gebeyehu et al. 2022), Wolaita (9.3%) (Asefa et al. 2023), Amhara, Oromia and Southern Nations, Nationalities, Peoples' Region (SNNP) (19.7%) (Bedassa et al. 2023), Oromia (20%) (Gume et al. 2023), Amhara (21.9%) (Yirsa and Tigistu 2025), and Tigrai (18.8%) (Abebe et al. 2014). Similarly, it was lower than other studies from Ecuador (37.5%) (Loor‐Giler et al. 2025) and Mongolia (14.26%) (Xie et al. 2025). On the contrary, the finding of the present study was a little bit higher than the previous studies reported from Ethiopia, including Sidama (1.85%) (Geinoro et al. 2025), Wolaita Sodo (1.89%) (Ayichew et al. 2024), Southern Ethiopia (1.2%) (Aliyo et al. 2022), Bishoftu‐Oromia (2%) (Temesgen et al. 2025). Likewise, it was higher than studies reported from Italy (0.1%) (Busani et al. 2005) and Spain (0%) (CABEDO et al. 2008). There are also other studies on bacterial contaminants of milk that did not report *Salmonella* in their findings from Ethiopia (Reda et al. 2014; Berhe et al. 2020) and Bangladesh (Khan et al. 2009). The main discrepancies of the results of *Salmonella* in cow milk among different regions and researchers might be due to difference in methodology such as use of small sample size and collection of different samples (raw or processed); isolation and identification methods such as difference in culture media, protocols, incubation periods and confirmation tests; Seasonal variation; difference in farm management, sanitation, infrastructure; and food handling, hand washing and milk boiling practices (Amenu et al. 2019; Yirsa and Tigistu 2025).

All *Salmonella* isolates were recovered exclusively from the bulk milks of dairy farms that do not have a separate milking house (p‐V = 1.00) and do not have specific hygiene protocols (p‐V = 1.00). These were found to be predictors of milk contamination based on the Fisher's exact test analysis. *Salmonella* was not isolated from the bulk milk of the dairy farms that have a separate milking house and specific hygiene protocols. This result indicated that poor personal hygiene and environmental hygiene may play a significant role in the incidence of *Salmonella* infection, although the absence of isolates among those reporting good hygiene practices highlights the protective effect of consistent sanitation behaviors. The adjusted odds of positive *Salmonella* were 5.44 times higher among those who don't wash udder before milking regularly (AOR = 5.44; 95% CI: 1.09–27.17; *p* = 0.039). This finding supports the previous studies that unwashed udder can raise the risk of *Salmonella* infection in raw milk consumers by suggesting that the bulk milk may be directly or indirectly contaminated by cow feces, the cow's skin and teat, milkers' hands during milking, and milking equipment (Deneke et al. 2022; Amenu et al. 2019; Geinoro et al. 2025; Yirsa and Tigistu 2025; Asefa et al. 2023). Similarly, the adjusted odds of *Salmonella* finding were 6.43 times higher among those who don't know that consumption of raw milk causes food‐borne illnesses (AOR = 6.43; 95% CI: 1.01–40.70; *p* = 0.048) which is in line with studies conducted in Ethiopia (Deneke et al. 2022) and India (Lindah et al. 2018). Similarly, *Salmonella* positivity has a statistically significant association with those who do not have knowledge about bacterial infections (p‐V = 0.030), which is in line with studies from Ethiopia (Lindah et al. 2018; Fufa Abunna and Bekele 2024; Sewunet et al. 2024). Because of the small number of positive isolates in our study, traditional logistic regression was not practical. Firth's logistic regression (penalized logistic regression) was employed to model the relationship between positivity and associated factors. Even in situations involving uncommon events, this technique minimizes small‐sample bias and yields estimates of the finite odds ratio. It is important to view the results as exploratory due to the limited number of incidents (Suhas et al. 2023).

Twelve antimicrobial agents from nine classes were used to evaluate six *Salmonella* isolates. Resistance to ampicillin was observed, and its prevalence was six (83.3%), which is comparable to studies from Ethiopia (Gebeyehu et al. 2022; Ayichew et al. 2024; Yirsa and Tigistu 2025) but higher than another study from Ethiopia (Temesgen et al. 2025). Similar resistance was reported for tetracycline, which was 5(83.3%). This was comparable to a study from Ethiopia (Yirsa and Tigistu 2025), but higher than other studies from Ethiopia (Temesgen et al. 2025; Ayichew et al. 2024). While we did not evaluate the sources of antibiotics used by the dairy farm workers to treat their lactating cows, a different study conducted in Northwestern Ethiopia found that dairy farm workers purchased the majority of medicines from private pharmacies. The two most commonly utilized antibiotics for their animals' treatment were ampicillin (72.5%) and tetracycline (76.9%) (Geta and Kibret 2021). As is likely to occur in our study area, such practical activities could be the primary causes of the rise in antibiotic resistance in Northwest Ethiopia. People who come into contact with dairy farms might not be fully aware that one of the factors contributing to the rise in antibiotic resistance is the use of antibiotics without prescription (Geta and Kibret 2021). The resistance patterns of *Salmonella* isolates to streptomycin were four (66.7%). This is similar to a study from central Ethiopia (Temesgen et al. 2025), but lower than another study from the Tigrai region of Ethiopia (Abebe et al. 2014) and higher than a study from Southern Ethiopia (Gebeyehu et al. 2022). The resistance patterns of *Salmonella* isolates to amoxicillin‐clavulanic acid were two (33.3%), which is similar to studies from Ethiopia (Yirsa and Tigistu 2025; Abebe et al. 2014). There was no isolate of *Salmonella* that showed resistance to ceftazidime, cefotaxime, and meropenem. The overall multidrug‐resistance (MDR) of *Salmonella* in this study was 3 (50%), which is lower than a study from Ethiopia (Temesgen et al. 2025; Ayichew et al. 2024).

## 5 Strength and Limitation

The collection of the sample directly from the dairy farms to identify the risk of milk contamination is considered as strength of the study. It has microbiological and epidemiological perspectives as it focuses on *Salmonella* isolation, antimicrobial susceptibility patterns, and risk factors. Although the isolates of *Salmonella* are small in number, the regional government can use this data as baseline information for surveillance of the dairy farms in the region. However, the presence of other species like poultry, biosecurity measures, availability of quality water, animal health status, parity, breed, lactation stage, and production system was not evaluated in the current study and may be considered as influential cofounders. Due to the small number of positive cases, we could not include additional variables in the multivariable regression model. As a result, there is a possibility of residual confounding as a limitation. Furthermore, the limited number of positive cases has led to wider confidence intervals, which may affect the precision of the estimates. Therefore, the findings of this study should be considered exploratory. There was also a financial limitation to carry out further analysis including serotyping and molecular characterization.

## 6 Conclusion and Recommendation

The prevalence of *Salmonella* in the present study was low, but its occurrence in the bulk milk may be considered a main risk factor for milk safety in the dairy farms. *Salmonella* showed high resistance to ampicillin and tetracycline, although all *Salmonella* isolates were susceptible to ceftazidime, cefotaxime, and meropenem. Half of the *Salmonella* isolates in the current study revealed multidrug resistance.

Lack of a separate milking house, lack of specific hygiene protocols, lack of knowledge about bacterial infections, lack of regular udder washing before milking, lack of Knowledge that the consumption of raw milk causes foodborne illness were considered as potential risk factors for the occurrence of *Salmonella* in the bulk milk of the dairy farms.

Our study forwarded its recommendation that all dairy farm workers should follow strict udder hygiene; enhance milking practices, and regularly conduct monitoring of foodborne infections including *Salmonella*, in the dairy farms. Moreover, this study recommends that health and agricultural authorities introduce regular food safety training to the dairy farm owners and workers in the study area. Food safety and hygiene specialists, veterinarians, public health professionals, and microbiologists should give emphasis introducing standards, protocols, and awareness‐creating manuals on how to prevent foodborne infections, including salmonellosis, in the Tigrai regional states of Ethiopia. This study also recommends that the scientific community should carry out large‐scale research related to foodborne pathogens in dairy farms of Tigrai, Northern Ethiopia, by using the outcome of this study as a baseline.

## Author Contributions

**Atsebaha Gebrekidan Kahsay:** conceptualisation, investigation, data curation, formal analysis, writing – original draft, editing and review. **Tsehaye Asmelash:** writing – editing and review. **Enquebaher Kassaye:** writing – editing and review.

## Conflicts of Interest

The authors declare no conflicts of interest.

## Data Availability

The data sets analyzed during the current study are available from the corresponding author.
